# Novel Cu/Zn Reinforced Polymer Composites: Experimental Characterization for Radiation Protection Efficiency (RPE) and Shielding Properties for Alpha, Proton, Neutron, and Gamma Radiations

**DOI:** 10.3390/polym13183157

**Published:** 2021-09-17

**Authors:** Ghada ALMisned, F. Akman, Waheed S. AbuShanab, Huseyin O. Tekin, Mustata R. Kaçal, Shams A. M. Issa, Hasan Polat, Meral Oltulu, Antoaneta Ene, Hesham M. H. Zakaly

**Affiliations:** 1 Department of Physics, College of Science, Princess Nourah Bint Abdulrahman University, Riyadh 11671, Saudi Arabia; gaalmisned@pnu.edu.sa; 2 Program of Occupational Health and Safety, Department of Property Protection and Security, Vocational School of Social Sciences, Bingöl University, Bingöl 12000, Turkey; fakman@bingol.edu.tr; 3 Central Laboratory Application and Research Center, Bingöl University, Bingöl 12000, Turkey; 4 Marine Engineering Department, Faculty of Maritime Studies and Marine Engineering, King Abdulaziz University, Jeddah 21589, Saudi Arabia; wabushanab@kau.edu.sa; 5 Medical Diagnostic Imaging Department, College of Health Sciences, University of Sharjah, Sharjah 27272, United Arab Emirates; 6 Medical Radiation Research Center (USMERA), Uskudar University, Istanbul 34672, Turkey; 7 Department of Physics, Arts and Sciences Faculty, Giresun University, Giresun 28100, Turkey; mustafakacal@hotmail.com; 8 Department of Physics, Faculty of Science, University of Tabuk, Tabuk 47512, Saudi Arabia; sh_issa@ut.edu.sa; 9 Physics Department, Faculty of Science, Al-Azhar University, Assiut 71452, Egypt; 10 Department of Architecture and Urban Planning, Vocational School of Technical Sciences, Bingöl University, Bingö 12000, Turkey; hpolat@bingol.edu.tr; 11 Department of Civil Engineering, Engineering Faculty, Atatürk University, Erzurum 25240, Turkey; mroltulu@atauni.edu.tr; 12 INPOLDE Research Center, Department of Chemistry, Faculty of Sciences and Environment, Physics and Environment, Dunarea de Jos University of Galati, 47 Domneasca Street, 800008 Galati, Romania; 13 Institute of Physics and Technology, Ural Federal University, 620002 Ekaterinburg, Russia

**Keywords:** brass composite, gamma-ray, charged particle, neutron, radiation shielding

## Abstract

In this study, brass (Cu/Zn) reinforced polymer composites with different proportions of brass powders were fabricated. Different types of nuclear shielding parameters such as mass and linear attenuation coefficients, radiation protection efficiency, half and tenth value layers, and effective atomic number values were determined experimentally and theoretically in the energy range of 0.060–1.408 MeV in terms of gamma-ray shielding capabilities of fabricated polymer composites. A high Purity Germanium detector (HPGe) in conjunction with a Multi-Channel Analyzer (MCA) and twenty-two characteristic gamma-ray energies have been used in the experimental phase. In addition, the exposure and energy absorption buildup factors of reinforced Cu/Zn composites were calculated, and relative dose distribution values were computed to verify them. Proton mass stopping power (Ψ_P_), proton projected range (Φ_P_), alpha mass stopping power (Ψ_A_), and alpha projected range (Φ_A_) parameters, which indicate the interactions of the produced composites with charged particle radiation, were investigated. Fast neutron removal cross-section (Σ_R_) results were determined to give an idea in terms of neutron shielding. According to the obtained results, it is reported that the CuZn20 coded sample’s ability to attenuate gamma-ray and charged particle radiation is more efficient than that of other prepared composites. A CuZn05 coded sample was found to be more suitable for neutron shielding capability.

## 1. Introduction

Technological advancements enabled the invention and ongoing improvement of a wide variety of equipment. This technological revolution has impacted numerous applications ranging from medical procedures to industrial advances during the last several years. Among emerging technologies, radiation sciences and its many applications continue to be a popular subject and are undergoing daily worldwide development. While the utilization of ionizing radiation is critical in essential applications that impact human life, such as medical diagnostics and medical radiation therapy, imprudent use of radiation, like any other activity, may have a significant effect on living biological structures DNA and materials. Therefore, the term ALARA (As Low As Reasonably Achievable) should always be considered in terms of radiation safety. This concept demands that, to the extent that it is practically possible, radiation protection measures promote the lowest radiation exposure duration and maximum distance between the public and radiation sources. Additionally, this concept implies the selection of the most suitable shielding material to limit exposure to the source. One of the critical variables here is the type and energy of the radiation released by the source, which will dictate the shielding material and design needed. It is worth noting that the most often utilized kind of radiation in medical and industrial applications is electromagnetic waves, sometimes known as X-rays or gamma-rays. Due to their excellent characteristics against X-rays and gamma-rays ionizing radiation, lead (Pb) and lead-based shields have been conventional components regarded as main shielding materials. However, lead (Pb) and lead-based shielding materials have a number of significant drawbacks, including toxicity, lack of transparency, and unsuitability for long-term use [1]. Consequently, the discovery and application of alternate radiation shielding materials has emerged as a prominent research subject in recent years [2,3,4,5,6]. The main goal of these materials is to keep radiation levels as low as possible. Additionally, it should be environmentally friendly throughout the production, use, and ultimate disposal phases and affordable and durable. Numerous studies have been conducted in recent years to determine the radiation shielding properties of glass materials [7,8,9], alloys [10,11,12], and composite materials [13,14] against ionizing radiation. A composite material, which is a general term, is often described as a substance comprised of two or more substantially distinct component materials with specific physical or chemical characteristics. It is worth noting that composite materials’ physical and chemical characteristics are often distinct from those of their components. Along with the composite’s basic structure, the kind and quantity of filler material used to strengthen the composite is critical to consider. Various experiments have been conducted before to determine the efficacy of various filler types and filler amounts in composite materials against ionizing radiation. The performance of polymer composites reinforced with BaTiO_3_ and CaWO_4_ fillers was studied by Akman et al. [15]. Their findings indicated that increasing the quantity of BaTiO_3_ and CaWO_4_ reinforcement improves the gamma-ray shielding effectiveness of polymer composites synergistically. Al-Dhuhaibat [16] has investigated the gamma-ray shielding characteristics of several epoxy polymers doped with cement, lead, iron, and aluminum. The findings indicated that Fe-filled composite samples behaved differently when exposed to gamma-rays produced by various kinds of point radioactive isotopes. The ongoing studies and their promising findings have encouraged us to fabricate some special types of polymer composites to investigate their nuclear radiation shielding properties. Accordingly, five brass (Cu/Zn) reinforced polymer composites encoded CuZn00, CuZn05, CuZn10, CuZn15, and CuZn20 were synthesized as part of an ongoing effort to develop effective and alternative shielding materials for ionizing radiation facilities. We hypothesised that these regular variations of filler contribution in the polymer composite might affect the characteristic behaviours against different types of nuclear radiation such as gamma-ray, neutron, alpha, and proton. Different types of phases as working flow have been planned as follows.

***Phase 1*:** sample preparation
***Phase 2*:** experimental gamma-ray transmission studies
***Phase 3*:** experimental studies of radiation protection efficiency (RPE)
***Phase 4*:** Monte Carlo simulations of nuclear radiation shielding properties

Experimental and advanced Monte Carlo simulation studies will be linked in terms of provided outcomes to assess the overall characterization process. The findings from this broad study may be used to further research next-generation, energy-efficient, and environmentally friendly composite materials and their application in radiation facilities.

## 2. Materials and Methods

Table 1 summarizes the chemical characteristics and mass densities of manufactured composite samples. The method of preparing composites in detail will be described in subsequent parts of this work. This study evaluated the mass attenuation coefficients (MAC) of manufactured composites in an experimental transmission setup using twenty-two different gamma-ray energies and a High Purity Germanium (HPGe) detector (Nel electronics, Ortec, TN, USA) We calculated the relative dose distribution (RDD) values and their behavior at various distances at 40 mean free path (mfp) to validate the derived shielding parameters. Finally, we assessed the radiation protection efficiency (RPE) of manufactured composites using experimental data. Brass reinforced composite samples encoded CuZn00, CuZn05, CuZn10, CuZn15, and CuZn20 were reported with material densities ranging from 1.1881 g/cm^3^ to 1.3649 g/cm^3^. The gamma-ray transmission properties were also simulated using the general-purpose Monte Carlo code MCNPX (version 2.4.0) [17].

### 2.1. Sample Preparation

Five different brass (Cu/Zn) reinforced polymer composites (Turkuaz Polyester, Turkuaz brand, Kocaeli, Turkey) were obtained by using:

(a) Unsaturated polyester resin, as binder,
(b) Methyl ethyl ketone peroxide (MEKP), as hardener,
(c) Cobalt Octoate-6% (Co-6) as accelerator, and
(d) Brass powders as filler.

**Figure 1:**
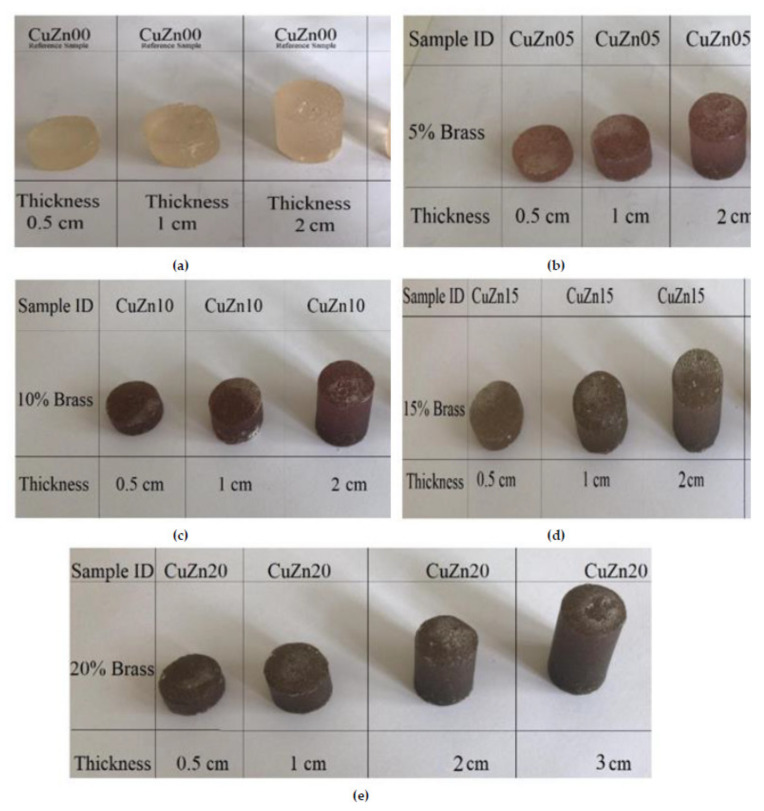
Fabricated brass composites: (**a**) unreinforced (CuZn00) reference polymer composite samples, (**b**) 5% brass reinforced (CuZn05) polymer composite samples, (**c**) 10% brass reinforced (CuZn10) polymer composite samples, (**d**) 15% brass reinforced (CuZn15) polymer composite samples, and (**e**) 20% brass reinforced (CuZn20) polymer composite samples.

**Figure 2:**
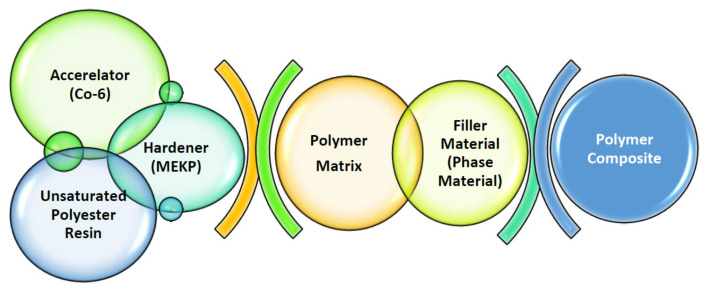
Production scheme of composite materials.

Carefully weighted amounts of binder and filler were prepared. The amount of filler was determined as approximately 5%, 10%, 15%, and 20%, in weight, of the amount of binder. The filler was homogenized in a mixer for one minute. Then, a mixture of binder and accelerator obtained by mixing for 1 min was poured on the filling material in the mixer, and they were mixed for 3 min to ensure homogeneity. Finally, the hardener was added to this mixture and mixed for 1 min. Mixing steps were applied as a standard for each series. The initiation of the polymerization process and the increase in reaction speed were carried out with the help of hardener (MEKP) and accelerator (Co-6), respectively. The composite production was completed by allowing the liquid sample to be cured in molds with a radius of 1 cm and a thickness of 0.5, 1.0, 2.0, and 3.0 cm (See Figure 1). Additionally, Figure 2 illustrates the manufacturing process.

### 2.2. Experimental Characterization of Gamma-Ray Transmission Parameters

To estimate the mass attenuation coefficients of brass (Cu/Zn) reinforced polymer composites, the shielding characteristics of the composites were studied utilizing a High Purity Germanium detector (HPGe) in conjunction with a Multi-Channel Analyzer (MCA). The dimensional properties of the utilized detector crystal of HPGe can be listed as below.

Diameter: 7 cm
Crystal length: 2.5 cm
Diameter of each lead collimator window: 1 cm
thicknesses of each lead collimator window: 1 cm.

**Figure 3:**
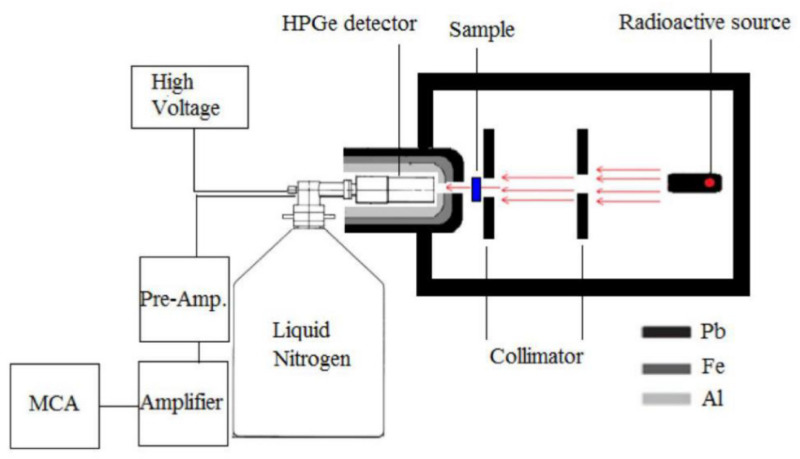
Narrow beam transmission geometry for experimental mass attenuation coefficient calculations.

They were located at 20 cm and 50 cm from the detector front wall. The distance source-detector was 80 cm. The sample was located at 10 cm from the detector. The general appearance of experimental setup geometry with used devices is shown in Figure 3. 

The following radioisotopes ^22^Na, ^54^Mn, ^57^Co, ^60^Co, ^133^Ba, ^137^Cs, ^152^Eu, and ^241^Am have generated twenty-two different gamma-ray energies varied from 0.060 MeV to 1.408 MeV. Further details about used radioactive isotopes can be found in literature elsewhere [18]. In this study, software from MAESTRO (Nel electronics, Ortec, TN, USA) was utilized to investigate the photo-peaks found during the data-acquisitions [19,20]. In addition, peak areas were determined by *Origin7.5* code (demo version) (OriginLab Corporation, Northampton, MA, USA)by using the least-square fitting method. To determine the value and uncertainties of experimental attenuation mass coefficients we relied on Equation (1)

(1) $$I=I_{0}\;exp\left({µ}_{m}\;\rho \;x\right)\;,\;\Delta {µ}_{m}=\frac{1}{x\rho }\sqrt{{\left(\frac{\Delta I}{I}\right)}^{2}+{\left(\frac{\Delta I_{0}}{I_{0}}\right)}^{2}+{\left(ln\left(\frac{\Delta I}{I}\right)\right)}^{2}\;{\left(\frac{\Delta \left(x\rho \right)}{x\rho }\right)}^{2}}$$

where *I_0_* and *I* are the area of the photo peak without and with a sample, respectively, *µ_m_* is the mass attenuation coefficient, ρ stands for the sample density, and *x* represents the sample thickness in front of the beam.

### 2.3. Shielding Parameters

The mass attenuation coefficient (${µ}_{m}$) may be determined using several numerical simulations and database methods, including WinXcom, MCNP, Xmudat, and Geant4. Hereby, all these methods are based on the mixture rule as [21]:

(2) $${µ}_{m}=\frac{µ}{\rho }=\frac{N_{A}}{M}\;\sigma_{tot}=\underset{k}{\sum }W_{k}\;{\left(\frac{µ}{\rho }\right)}_{k}\;$$

In the Equation (2), µ is the Linear Attenuation Coefficient (LAC), *N_A_* is the Avogadro number, *M* and *σ_tot_* are the atomic mass and total microscopic cross section of the sample, and *W_k_* is a weight fraction of the *k^th^* element in the fabricated brass composite sample. Derived from this basic quantity, mfp (mean free path) and HVL (half value layer), and TVL (Tenth Value Layer) can be calculated as shown in Equations (3) and (4). As observed, HVL and TVL are proportional to mfp. The physical meaning of mfp is the average distance a gamma ray of a given energy traverses in a medium until it interacts with the medium for the first time. HVL and TVL are the average distances required for the incident flux of gamma rays to decrease its intensity by a half and a tenth, respectively. It is worth noting that removing the gamma rays from the incident beam does not necessarily imply that it will no longer contribute to the dose delivered by the radiation source to the patient or public, as secondary radiation might still reach them.

(3) mfp=1/μ  

(4) $$HVL=\frac{ln2}{\mu }\;\;\;;\;\;\;\;\;TVL=\frac{ln10}{\mu }\;$$

*Z_eff_* (effective atomic number) is another important parameter to consider when assessing gamma radiation shielding, since electromagnetic radiation is mostly dispersed by electrons of the atomic shell. *Z_eff_* has far more information than the electron density, as it also depends on the gamma radiation energy, which is why it is an interesting parameter in Radiation Shielding Design. *Z_eff_* can be determined for each composite sample by utilization of Equations as follows [22]:

(5) $$Z_{eff}=\frac{\sum_{j}\;f_{j\;}A_{j}\;{\left({µ}_{m}\right)}_{j}}{\sum_{j\;}f_{j\;}\;\frac{A_{j}}{Z_{j}\;}{\left({µ}_{m}\right)}_{j}}$$

In Equation (5), *f_j_* stands for the mole fraction of species j, which has molar mass *A_j_,* atomic number *Zj* and mass attenuation coefficient ${\left({µ}_{m}\right)}_{j}$. 

The calculation of *Z_eff_* involves the contribution of all processes which can occur during the gamma-atom interaction. Depending on the gamma radiation energy, the Peak to Compton ratio changes and the probability that each of these effects occurs. Hence, the difference between these parameters magnifies the sensitivity of these phenomena by the sample. The Effective Buildup Factor (EBF) and the Effective Absorption Buildup Factor (EABF) somehow take into account the contribution of those secondary radiations mentioned above when discussing mfp. The EBF estimates the ratio between the contribution of all gamma detected (primary and secondary radiation), and the contribution of those gammas that are detected which have not had any interaction between the radiation source and the detector (primary radiation). The EABF takes into account how much energy was deposited in the material by both primary and secondary radiation, with respect to how much energy was deposited in the detector only by primary radiation. EBF and EABF are critical metrics for determining the radiation shielding effectiveness of the absorber material environment under investigation. These parameters, EBF and EABF, provide detailed information on the quantity of photons, their intensity, energy flow, and dose. EBF and EABF of brass reinforced polymer composites were calculated using the well-established Geometric Progression (G-P) fitting technique [23]. To estimate buildup factors of composites reinforced with brass, the equivalent atomic numbers (*Z_eq_*) were determined. The *Z_eq_* values can be calculated based on Equation (6) as follows:

(6) $$Z_{eq}=\frac{Z_{1}\left(logH_{2}-logH_{x}\right)-Z_{2}\left(logH_{1}-logH_{x}\right)}{logH_{2}-logH_{1}}$$

Equation (6) should be calculated at a given energy and sample. The quantity of *H_x_* is the ratio between Compton attenuation and total attenuation (*(µ/ρ)_Comp_/(µ/ρ)*) for the energy and sample under consideration. *H_2_* and *H_1_* are the corresponding ratios for two successive atomic numbers *Z_1_* and *Z_2_ = Z_1_+1*, so that H_x_ lies between *H_1_* and *H_2_*. 

We utilized *Z_eq_* values to obtain the G-P fitting parameters by the Equations (7)–(9). Thus, at last, the buildup factors of composites reinforced with brass were successfully determined.

(7) $$B\left(E,X\right)=1+\frac{\left(b-1\right)\left(K^{x}-1\right)}{K-1}\;\;\;\;\;\;\mathrm{for}\;K\neq 1$$

(8) $$\left(E,x\right)=1+\left(b-1\right)x\;\;\;\mathrm{for}\;K=1$$

where

(9) $$K\left(E,x\right)=cx^{a}+d\frac{\tanh\left(\frac{x}{x_{k}}-2\right)-\tanh\left(-2\right)}{1-\tanh\left(-2\right)},\;x\leq 40MFP\;$$

*x* denotes the distance between the source and detector in Equations (7)–(9). At 1 mfp, the EBF is represented as b. The *K* (*E, X*) factor denotes dosage multiplication. The capacity of brass reinforced polymer composites to attenuate fast neutrons was investigated in this article. Therefore, the effective removal cross section (Σ_R_/ρ, in cm^2^/g) values neutron shielding part were determined. The determination process of Σ_R_/ρ and heavy charged particles with all detail can be found in our previous studies [24]. On the other hand, the term radiation protection efficiency (RPE) is a critical metric to consider when evaluating the attenuation qualities of potential shielding materials. Equation (10) may be used to calculate this parameter [25].

*RPE (%) = (1 – I/I_0_) × 100* (10)

where *I* denotes photon counts that have been attenuated, and *I_0_* denotes photon counts that have not been attenuated. Moreover, the SRIM Monte Carlo code was used to estimate the fundamental shielding properties for heavy-charged ions as follows.

Proton mass stopping power/PSP (Ψ_P_)
Proton projected range/PPR (Φ_P_)
Alpha mass stopping power/ASP (Ψ_A_)
Alpha projected range/APR (Φ_A_)

These parameters of the manufactured composite samples were extensively determined in addition to gamma-ray and neutron interactions [26]. Our previous study [1] has detailed information on computations and technical details.

### 2.4. Monte Carlo Simulations

**Figure 4:**
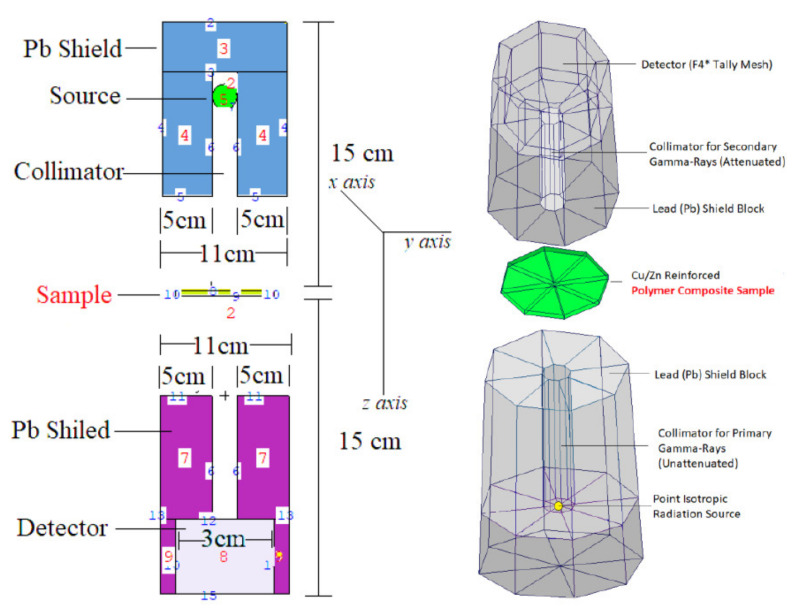
MCNPX simulation setup used for gamma-ray transmission simulations (a direct screenshot from the MCNPX Visual Editor *VE X_22S*).

To verify the experimental findings, MCNPX (version 2.7.0) (Los Alamos National Laboratory, Los Alamos, NM, USA)was also utilized in this study. This code is very flexible, has been thoroughly tested and validated, and has been used to develop and verify additional assessments involving photon, neutron, and charged particle transport. The code may need two modules as input; the first specifies the system’s shape in as much detail as the user desires, as well as the densities of each material. The second one includes the material composition, a description of the radiation source, the evaluations required to produce the required outputs, and the number of histories to compute to reach significant accuracy. Additionally, the program makes use of libraries of data detailing cross sections and other nuclear data, which are required to reproduce all of the particle transport processes. Figure 4 depicts the whole simulation setup obtained from MCNPX Visual Editor, complete with specified simulation equipment for calculating the attenuation coefficients. Between the source of isotropic point radiation and the detecting field, a polymer composite as gamma-ray attenuator has been placed. Additionally, two significant Lead (Pb) blocks have been built to absorb scattering gamma rays, which may improve detection consistency. Finally, each polymer composite sample was subjected to a total of 10^8^ particle tracks with different photon intensities (Number of History). After all simulations were completed, the MCNPX output had a relative error rate of less than 1%. All the MCNPX simulations were performed on a Lenovo^TM^ ThinkStation620 equipped with a Ryzen^TM^ Threadripper^TM^ Pro 3995WX CPU (2.7GHz, 64 Cores, 256MB Cache). 

## 3. Results and Discussion

**Figure 5:**
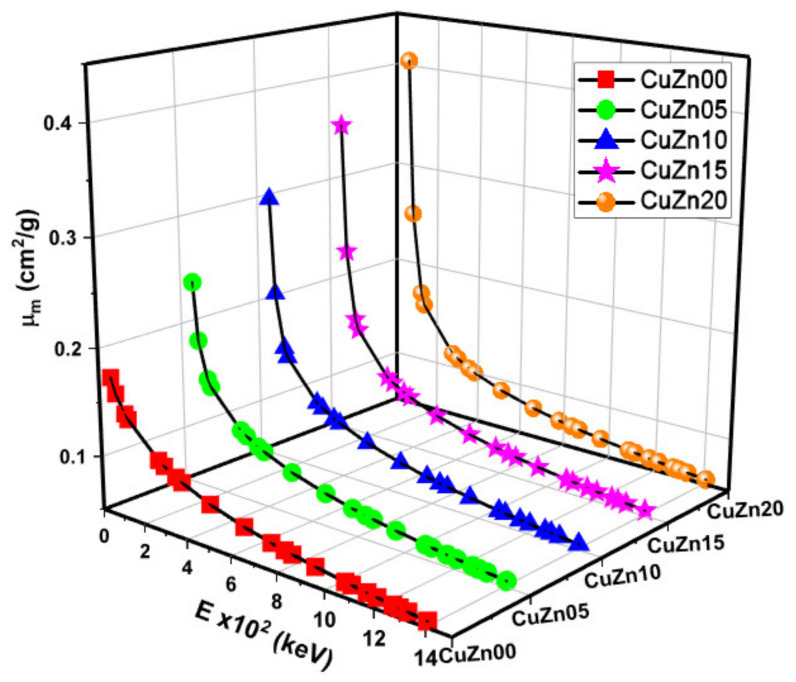
Mass attenuation coefficient (*μ_m_*) values as a function of photon energy and Cu-Zn content of samples.

The chemical contents and densities of the materials are given in Table 1. The $\mu_{m}$ (MAC) values between 0.060 and 1.408 MeV energy region were measured by experimental and Monte Carlo methods. Figure 5 and Table 2 show the obtained experimental, Monte Carlo simulation, theoretical mass attenuation coefficients, and corresponding energy values. From the figure and table**,** we observe that the $\mu_{m}$ values decrease with increasing energy, which is expected, as the higher the gamma-ray energies have higher penetration properties. Additionally, we found that the samples with greater brass content have higher MAC values, implying that the shielding ability is improved. This is also a natural occurrence, since brass components increase the mass of the substance, which is mostly composed of Copper and Zinc. If the mass attenuation coefficients of the produced composites are compared with those of lead (Pb) and tungsten (W), which are commonly used in radiation shielding; At 662 keV, the mass attenuation coefficients of CuZn00, CuZn05, CuZn10, CuZn15, and CuZn20 are 23.8%, 24.4%, 24.9%, 25.3% and 25.8% lower, respectively, than that of Pb with a mass attenuation coefficient 0.0999 cm^2^/g [27]. At the same energy, the mass attenuation coefficients of CuZn00, CuZn05, CuZn10, CuZn15, and CuZn20 are 15.6%, 16.2%, 16.6%, 17.1%, and 17.5% lower, respectively, than that of W with a mass attenuation coefficient 0.0933 cm^2^/g [28]. Ahmed et al. [29] developed flexible silicone-based composites with tungsten additives at different ratios for use in radiation shielding. The highest percentage (88.1%) of tungsten added flexible silicone/tungsten composite has a mass attenuation coefficient of 0.0961 cm^2^/g at 662 keV, and the CuZn20 coded 20% brass added composite has a 21% lower mass attenuation coefficient than this sample. Alsayed et al. [30] investigated the radiation shielding properties of high-density polyethylene (HDPE) based composites with zinc oxide (ZnO) at different ratios. At 662 keV, the highest ZnO doped (40%) composite has a mass attenuation coefficient of 0.065 cm^2^/g. When the mass attenuation coefficient of this sample is compared with that of the CuZn20 coded sample, the CuZn20 coded sample has a 22.2% higher mass attenuation coefficient than the other. In other words, the CuZn20 coded sample is a better radiation shielding material than the 40% ZnO doped and HDPE-based composite. 

**Figure 6:**
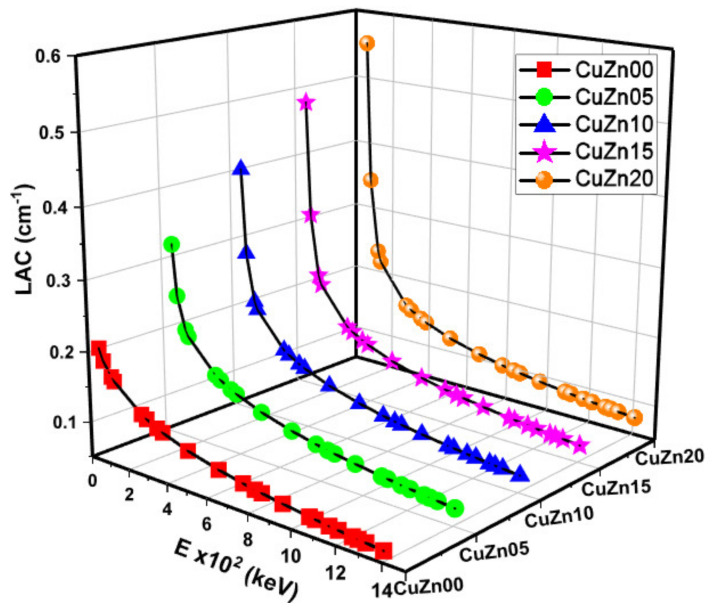
Linear attenuation coefficient (LAC) values as a function of photon energy and Cu-Zn content of samples.

**Figure 7:**
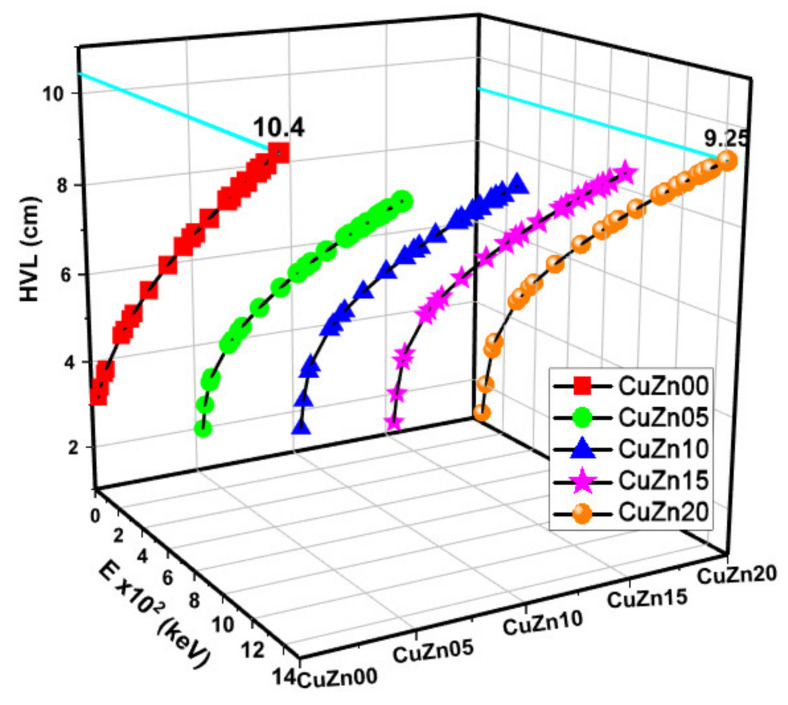
Half value layer (HVL) values as a function of photon energy and Cu-Zn content of samples.

**Figure 8:**
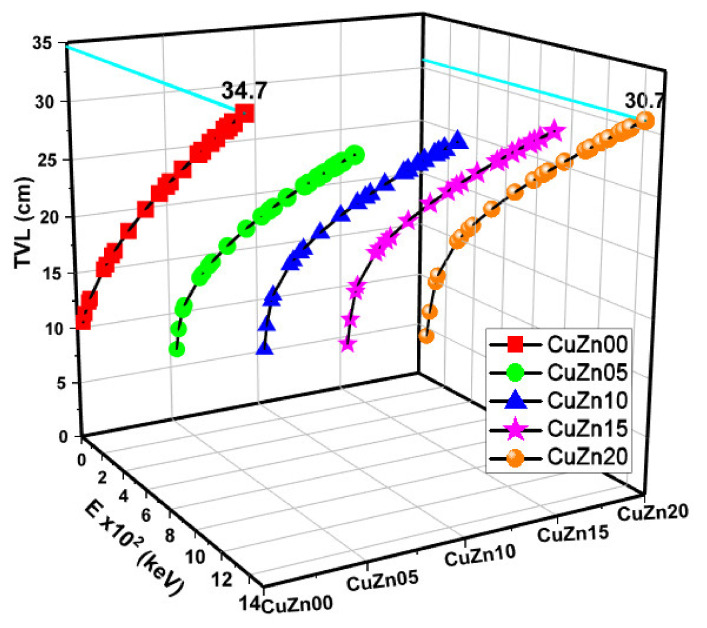
Tenth value layer (TVL) values as a function of photon energy and Cu-Zn content of samples.

**Figure 9:**
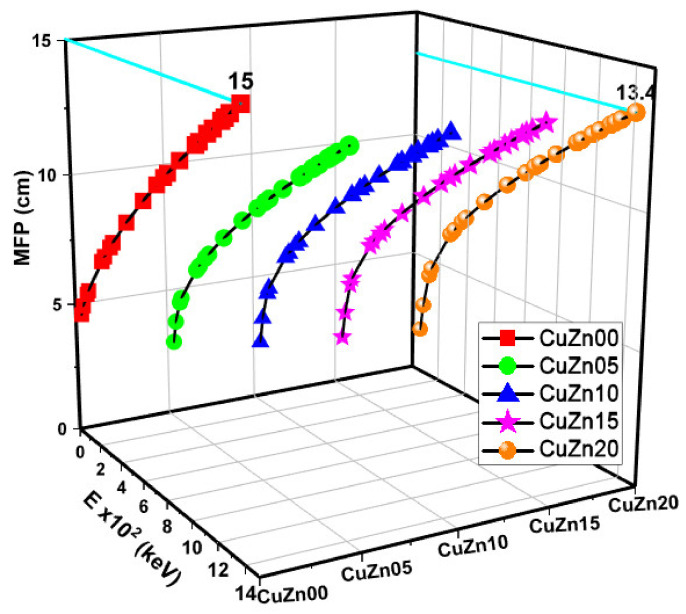
Mean free path (MFP) values as a function of photon energy and Cu-Zn content of samples.

The LAC values, proportionate to the MAC, are shown in Figure 6. At all energy levels, CuZn20 exhibits higher LAC values than other materials. The relationship between HVL-TVL and mfp is inversely proportional to the relationship between MAC and mfp. As anticipated, Figure 7, Figure 8 and Figure 9 show that these lengths grow in length when the gamma energy rises and increase in size as the material density decreases. As a general observation, about 30 cm of these composites is required to decrease the intensity of un-collided high energy gammas to a tenth, while a shield of 10 cm thickness would suffice for low energies. We argued earlier that polymer composite density would increase as brass content increases in the structure. However, the effective electron density experienced by the incident gamma-ray beam will depend on the energy of the radiation because different phenomena can take place, changing the resultant transmitted beam. 

**Figure 10:**
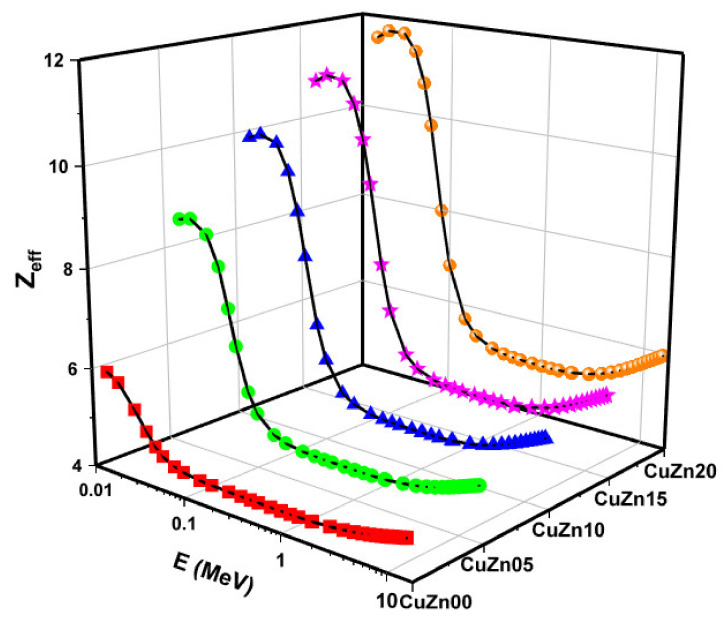
Effective atomic number (Z_eff_) values as a function of photon energy and Cu-Zn content of samples.

Figure 10 illustrates the effective atomic number Z_eff_ for each composite as a function of the incident gamma energies. For each energy, we notice that Z_eff_ increases with the content of brass, as argued earlier. However, we realized that for a given composite, at lower energies, Z_eff_ is highest, decreasing out about 0.2 MeV, then Z_eff_ is flat until energies of 1 MeV are considered, at which point Z_eff_ begins to increase again. This behaviour can be related to the regions where Photoelectric, Compton Scattering, or Pair Production are dominant. 

**Figure 11:**
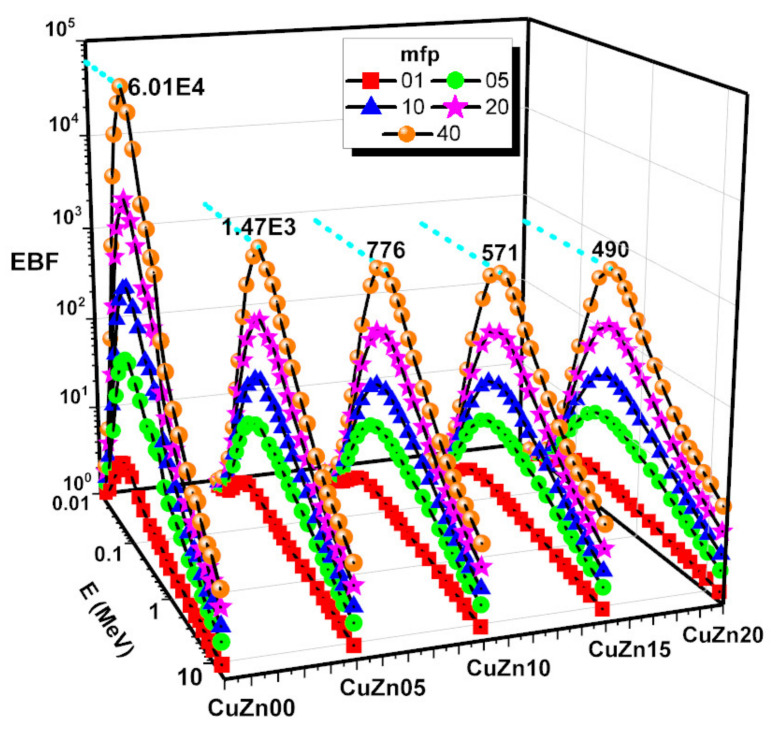
Exposure buildup factor (EBF) against photon energy of samples at 1, 5, 10, 20 and 40 mfp.

**Figure 12:**
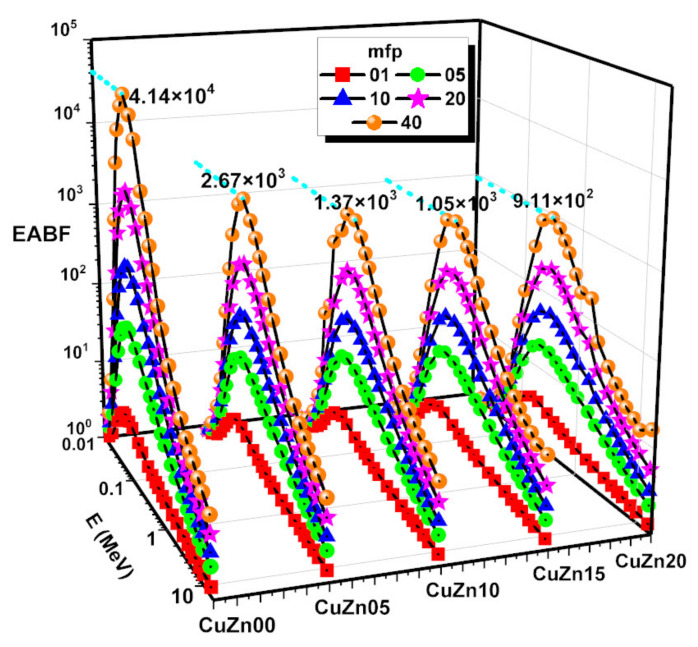
Energy absorption buildup factor (EABF) against photon energy of samples at 1, 5, 10, 20 and 40 mfp.

By using the G–P fitting approach (Table 3, Table 4, Table 5, Table 6 and Table 7), the EBF and EABF values were calculated at energies as high as 15 MeV, and penetration depth of 1–5–10–20–40 mfp. The calculated EBF and EABF values of CuZn00, CuZn05, CuZn10, CuZn15, CuZn20 are given in Figure 11 and Figure 12**.** For each composite, we found that at low energies, when photoelectric effect is dominant, EBF and EABF are low. In comparison, EBF and EABF values rise in the mid-energy range, where Compton Scattering is the main interaction between the incoming gamma-ray and the material. This is because no secondary gamma radiation is released directly, and although the electron absorbing the gamma energy may ionize or excite the atoms along the route, the secondary radiation emitted should be readily reabsorbed by the surrounding atoms. For intermediate energies, EBF and EABF reach a maximum consistent with the maximum of Compton Effect probability. The Compton Effect generates secondary gamma radiation naturally, which adds to the build-up. At high energies, the likelihood of the Compton Effect diminishes, and only positron annihilation caused by pair formation may contribute to the build-up. When we compare the various composites, we see that the lower the brass percentage, the greater the build-up, which is consistent with the more effective shielding that would come from a higher electrical density. As can be seen in Figure 11 and Figure 12, the greatest values of EBF and EABF for each chemical at 40 mfp have been noted. EABF is higher than EABF for every composite except for CuZn00. This is consistent with the idea that in the absence of brass the secondary photons produced by Compton Effect have lower chances to be attenuated in the material, compared to the rest of the compounds. 

**Figure 13:**
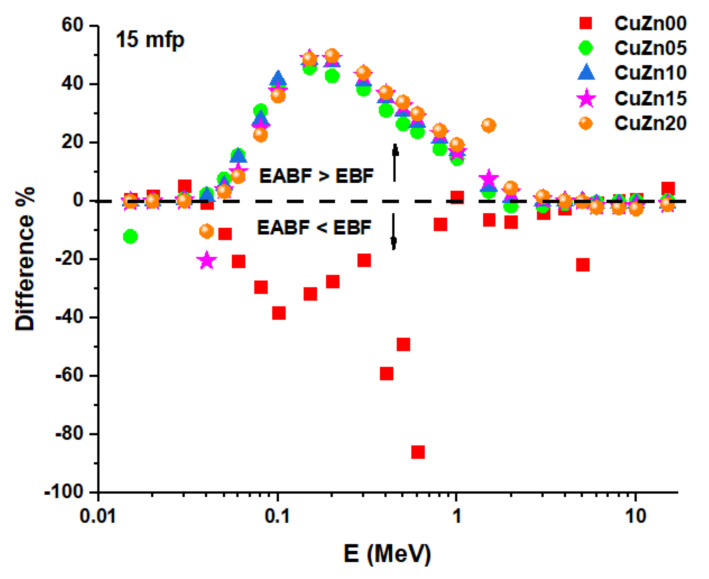
Difference between energy absorption buildup factor (EABF) and exposure buildup factor (EBF) of samples at 15 mfp.

**Figure 14:**
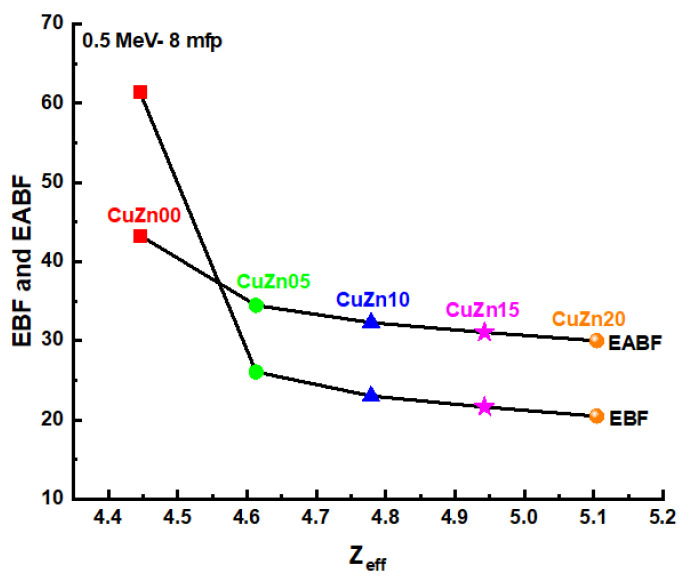
Variation of energy absorption buildup factor (EABF) and exposure buildup factor (EBF) against effective atomic number (Z_eff_) of samples at 8 mfp and 0.5 MeV.

In Figure 13, this comparison is depicted for samples at 15 mfp, where only for CuZn00 it results in EABF smaller than EBF. While Figure 14 illustrate the Variation of energy absorption buildup factor (EABF) and exposure buildup factor (EBF) against the effective atomic number (Z_eff_) of samples at 8 mfp and 0.5 MeV. 

**Figure 15:**
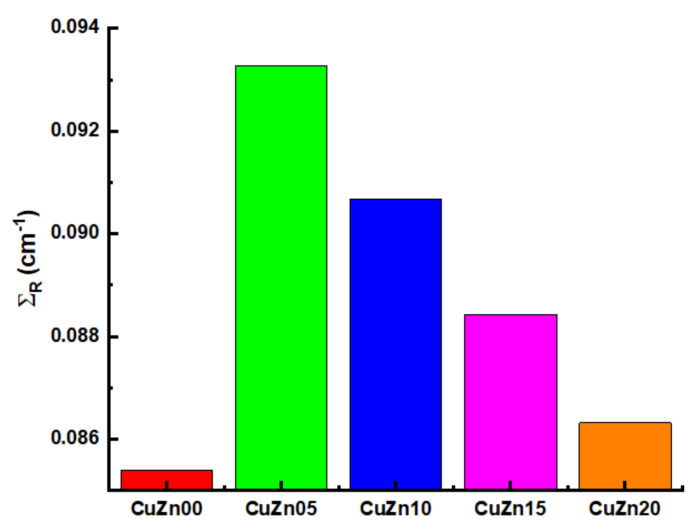
Fast neutron removal cross sections (Σ_R_) values for the selected samples.

The Σ_R_ values for the sample are given in Figure 15. Higher values of the fast neutron removal cross-section (Σ_R_) in the CuZn-composite samples, with a maximum in mixture CuZn05, are related with the higher composite densities and amounts of H, C, and O, with the highest in the CuZn05 sample. The concentrations of Cu and Zn have no effect on the Σ_R_ dependence for the considered CuZn-composites. The highest ∑_R_ value was reported for the CuZn05 sample as 0.0931 cm^−1^. Next, APR, ASP, PPR, PSP values pf CuZn00, CuZn05, CuZn10, CuZn15, and CuZn20 composite samples were calculated using the SRIM code. According to common assumptions, significant energy is lost in the composite samples medium through PMSP and AMSP. 

**Figure 16:**
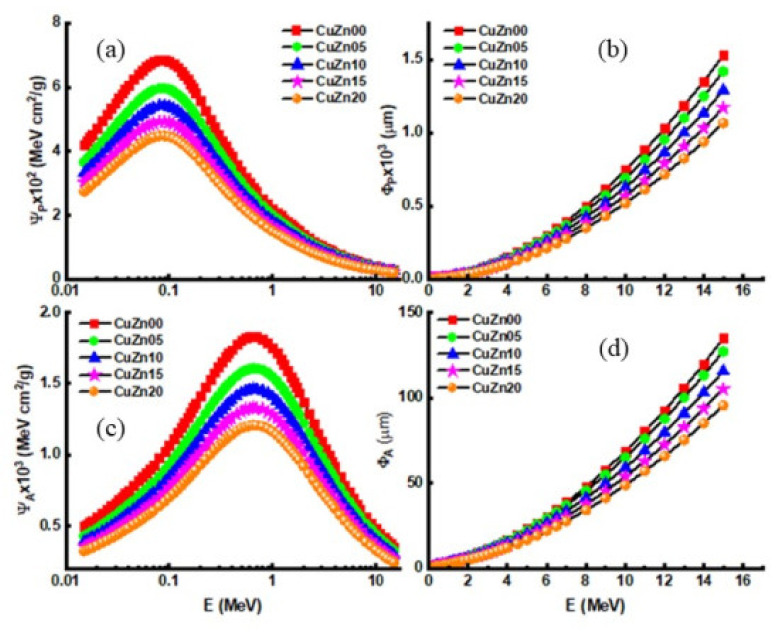
(**a**) Proton mass stopping power (Ψ_P_), (**b**) proton projected range (Φ_P_), (**c**) alpha mass stopping power (Ψ_A_) and (**d**) alpha projected range (Φ_A_) of samples.

Figure 16 reports the changes in the PMSP and AMSP values for CuZn00, CuZn05, CuZn10, CuZn15 and CuZn20 composite samples with varying energy levels. As seen in Figure 16, PMSP values rise as energy increases up to 0.07 MeV. Similarly, with rising energy up to 0.7 MeV, AMSP values also increase, as shown in Figure 16b. Moreiver, Figure 16a,b shows that AMSP and PMSP values decline depending on the increase in Cu and Zn concentration. Per the results, the lowest possible AMSP-PMSP in the energy range between 0.015–15 MeV is possessed by the CuZn20 sample. This situation mainly occurs because CuZn20 is the sample with the largest atomic numbers (Cu = 29 and Zn = 30) and the highest density (1.3649 g/cm^3^). Figure 16c,d show that the minimum PPR and ARP values belong to the CuZn20 sample, serving much better in terms of alpha and proton shielding than the others. The term RPE is a helpful metric for comparing the original and attenuated gamma source counts. This study evaluated the RPE values of produced polymer composites at four different thicknesses (0.5, 1.0, 2.0, and 3.0 cm).

**Figure 17:**
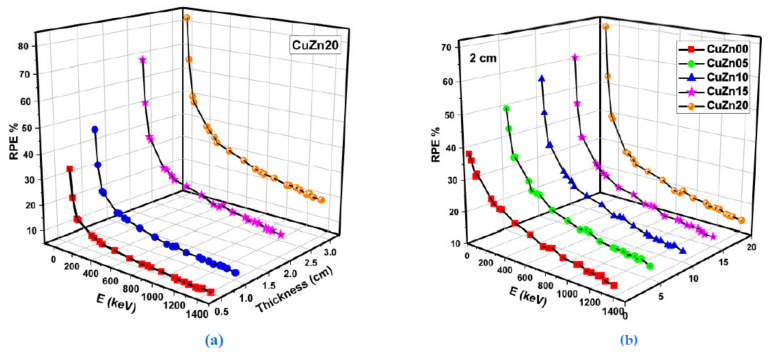
(**a**) Radiation protection efficiency (RPE) of CuZn20 sample at different thicknesses (**b**) Radiation protection efficiency (RPE) of fabricated composites at 2 cm.

Figure 17 illustrates the obtained experimental RPE values. As shown in Figure 17a, the RPE values of the superior composite polymer encoded CuZn20 increased linearly with material thickness. On the other hand, Figure 17b shows that increasing brass reinforces the amount in the composite structure, increasing the RPE at 2 cm material thickness. The purpose of this study was to determine the various effects of increasing the quantity of brass filler in manufactured polymer composites on their nuclear radiation shielding characteristics. Apart from the cross sections for effective removal cross-section for fast neutrons, the findings indicated that increasing the quantity of brass filler has several effects on nuclear radiation shielding characteristics.

## 4. Conclusions

In the present study, gamma-rays, charged particles and neutron attenuation characteristics of the reinforced composites prepared with adding different amounts of brass powders were investigated. MAC, LAC, RPE, HVL, TVL, mfp and Z_eff_, which are gamma-ray attenuation parameters, were investigated in the energy range of 0.060–1.408 MeV. To observe the variation of RPE values with thickness, the measurements of samples with 0.5, 1.0, 2.0 and 3.0 cm thicknesses were carried out within the specified energy range. EBF and EABF values of these reinforced composites were calculated up to 15 MeV and 40 mfp penetration depth, and their RDD values were computed for control purposes. While Ψ_P_, Φ_P_, Ψ_A_ and Φ_A_ parameters in terms of charged particle-matter interactions were determined, the ∑_R_ parameter was calculated to investigate neutron attenuation properties. The following observations are reported from the determined parameters with the help of experimental, theoretical, or simulation codes.

It is seen that LAC, MAC, RPE, and Z_eff_ parameters decrease exponentially with increasing photon energy and increase with increasing filler concentration. In addition, it was observed that RPE values increase rapidly with increasing thickness in samples considered in different thicknesses.

It was observed that HVL, TVL and mfp values increase with increasing energy but decrease with increasing filler concentration. This means that these composites are better gamma-ray shielding material in the low energy region than high energies, and a smaller thickness material is required for gamma-ray attenuation when the filler concentration increases.

According to the results of EBF and EABF, it has been reported that in the medium energy region where the Compton scattering cross section is dominant, these parameters take maximum values, whereas in the low and high energy regions, where the photoelectric and pair production cross sections are dominant, respectively, EBF and EABF values have taken relatively low values. In addition, it is noted that these parameters decrease with increasing filler concentration.

It was observed that Ψ_P_ and Ψ_A_ parameters increase exponentially up to 0.07 MeV and 0.7 MeV energies, respectively, and decrease similarly after these energies, and decrease with increasing filler concentration. Φ_P_ and Φ_A_ parameters increase with increasing photon energy and decrease with increasing filler concentration.

∑_R_ values, which is another important parameter, decrease with increasing filler concentration. It has been observed from the ∑_R_ results that while the CuZn00 coded sample is a bad neutron shielding material, CuZn20 coded composite is a good neutron shielding material compared to the other composites.

In terms of gamma-ray and charged particle attenuation characteristics, it was observed that a CuZn20 coded sample is a good shielding material compared to other composites.

It was aimed in this study to show how the amount of additive affects the attenuation parameters in prepared composites with a filler concentration between 5% and 20% (in 5% steps). It was noted in this study that the increase of filler concentration in the composites contributes positively for all parameters, except the ∑_R_ parameter, in terms of radiation shielding characteristics. Therefore, these composites, which are recommended in terms of radiation shielding, can be preferred in nuclear power plants, radiation-related units of hospitals, and research laboratories due to their lightness, easy production, and easy formability advantages.

## Figures and Tables

**Table 1:** Samples codes, elemental compositions (wt%) and density (ρ) of samples.

Code	Co	C	H	O	Cu	Zn	ρ (g/cm^3^)
**CuZn00**	0.0347	59.9693	4.5867	35.4093	-	-	1.1881
**CuZn05**	0.0331	57.2083	4.3756	33.779	2.7307	1.8733	1.3436
**CuZn10**	0.0317	54.6902	4.183	32.2923	5.2211	3.5817	1.3496
**CuZn15**	0.0304	52.3845	4.0066	30.9309	7.5015	5.1461	1.3577
**CuZn20**	0.0291	50.2655	3.8446	29.6796	9.5973	6.5839	1.3649

**Table 2:** Experimental, theoretical and Monte Carlo mass attenuation coefficients (cm^2^/g) results of brass reinforced composites.

	CuZn00			CuZn05			CuZn10			CuZn15			CuZn20		
Energy (keV)	Experimental	WinXcom	MCNPX	Experimental	WinXcom	MCNPX	Experimental	WinXcom	MCNPX	Experimental	WinXcom	MCNPX	Experimental	WinXcom	MCNPX
59.54	0.1904	0.1887	0.1909	0.2534	0.2583	0.2591	0.3280	0.3215	0.3224	0.3613	0.3795	0.3804	0.4149	0.4325	0.4331
81.00	0.1622	0.1697	0.1701	0.1917	0.1974	0.1983	0.2193	0.2226	0.2231	0.2425	0.2457	0.2461	0.2642	0.2668	0.2671
122.06	0.1427	0.1502	0.1516	0.1536	0.1580	0.1584	0.1714	0.1651	0.1663	0.1702	0.1716	0.1724	0.1710	0.1775	0.1783
136.47	0.1473	0.1453	0.1465	0.1571	0.1507	0.1512	0.1514	0.1556	0.1561	0.1550	0.1602	0.1612	0.1656	0.1643	0.1645
276.40	0.1168	0.1150	0.1162	0.1114	0.1152	0.1128	0.1123	0.1154	0.1158	0.1113	0.1156	0.1163	0.1144	0.1157	0.1163
302.85	0.1149	0.1112	0.1117	0.1113	0.1112	0.1119	0.1082	0.1112	0.1117	0.1060	0.1113	0.1127	0.1154	0.1113	0.1118
356.02	0.1056	0.1045	0.1051	0.1052	0.1044	0.1048	0.1082	0.1042	0.1048	0.1088	0.1041	0.1059	0.1055	0.1040	0.1051
383.85	0.0976	0.1015	0.1019	0.1049	0.1013	0.1019	0.1058	0.1011	0.1014	0.1032	0.1009	0.1007	0.1019	0.1007	0.1014
511.00	0.0896	0.0903	0.0910	0.0913	0.0900	0.0914	0.0933	0.0897	0.0905	0.0876	0.0894	0.0899	0.0904	0.0891	0.0905
661.66	0.0842	0.0807	0.0815	0.0835	0.0803	0.0812	0.0832	0.0800	0.0822	0.0830	0.0797	0.0806	0.0795	0.0794	0.0799
778.90	0.0726	0.0749	0.0761	0.0767	0.0746	0.0750	0.0709	0.0742	0.0753	0.0774	0.0739	0.0741	0.0702	0.0737	0.0738
834.85	0.0754	0.0725	0.0734	0.0746	0.0722	0.0728	0.0723	0.0719	0.0725	0.0746	0.0716	0.0724	0.0692	0.0713	0.0710
867.38	0.0684	0.0712	0.0723	0.0697	0.0709	0.0711	0.0711	0.0706	0.0721	0.0694	0.0703	0.0713	0.0725	0.0700	0.0707
964.08	0.0646	0.0677	0.0682	0.0688	0.0674	0.0681	0.0640	0.0671	0.0683	0.0658	0.0668	0.0671	0.0644	0.0665	0.0661
1085.87	0.0649	0.0639	0.0643	0.0620	0.0636	0.0639	0.0654	0.0633	0.0638	0.0622	0.0630	0.0644	0.0635	0.0627	0.0633
1112.07	0.0609	0.0631	0.0635	0.0654	0.0628	0.0631	0.0594	0.0625	0.0631	0.0606	0.0622	0.0632	0.0609	0.0620	0.0624
1173.24	0.0598	0.0615	0.0626	0.0631	0.0611	0.0614	0.0625	0.0608	0.0617	0.0582	0.0606	0.0615	0.0613	0.0603	0.0615
1212.95	0.0594	0.0604	0.0610	0.0606	0.0601	0.0610	0.0624	0.0598	0.0609	0.0619	0.0595	0.0591	0.0575	0.0593	0.0606
1274.53	0.0559	0.0589	0.0593	0.0611	0.0586	0.0591	0.0554	0.0583	0.0591	0.0601	0.0580	0.0584	0.0605	0.0578	0.0581
1299.14	0.0564	0.0583	0.0587	0.0587	0.0580	0.0584	0.0589	0.0577	0.0581	0.0596	0.0575	0.0585	0.0597	0.0573	0.0561
1332.50	0.0547	0.0576	0.0579	0.0546	0.0573	0.0571	0.0591	0.0570	0.0576	0.0576	0.0567	0.0573	0.0582	0.0565	0.0577
1408.01	0.0581	0.0560	0.0564	0.0541	0.0557	0.0559	0.0539	0.0554	0.0558	0.0528	0.0552	0.0558	0.0541	0.0549	0.0551

**Table 3:** (EBF and EABF) G-P fitting coefficients (b. c. a. X_k_ and d) of CuZn00 sample.

E (MeV)	Z_eq_	EBF					EABF				
		b	c	a	X_k_	d	b	c	a	X_k_	d
0.015	6.75	1.2702	0.4903	0.1650	14.2795	−0.0825	1.2746	0.4948	0.1606	14.5342	−0.0782
0.020	6.76	1.6183	0.6189	0.1185	15.3538	−0.0579	1.6359	0.6143	0.1209	15.2516	−0.0594
0.030	6.78	2.8080	0.9073	0.0362	15.1029	−0.0296	2.9248	0.9081	0.0354	15.3190	−0.0277
0.040	6.79	4.2027	1.3703	−0.0679	13.6447	0.0265	4.1965	1.3627	−0.0659	13.8588	0.0245
0.050	6.78	5.3801	1.7358	−0.1247	13.9503	0.0539	5.0372	1.7192	−0.1218	14.0664	0.0518
0.060	6.79	5.8432	2.0245	−0.1626	13.8467	0.0746	5.1989	1.9912	−0.1579	13.9445	0.0709
0.080	6.84	5.6297	2.3435	−0.1989	13.4691	0.0908	4.9054	2.2640	−0.1887	13.6347	0.0825
0.100	6.80	5.3364	2.4076	−0.2000	14.3919	0.0867	4.5940	2.3045	−0.1879	14.5553	0.0782
0.150	6.89	4.0121	2.4953	−0.2128	14.1131	0.0956	3.7258	2.3187	−0.1906	14.4664	0.0767
0.200	6.91	3.4241	2.4317	−0.2100	13.4641	0.0905	3.3493	2.2089	−0.1804	14.7975	0.0761
0.300	6.96	2.9694	2.1529	−0.1812	14.0741	0.0763	2.8392	2.0589	−0.1683	14.2401	0.0669
0.400	5.96	2.8438	2.2142	−0.1941	13.4346	0.0817	2.6252	1.9291	−0.1536	14.8201	0.0632
0.500	5.96	2.6579	2.0255	−0.1730	14.1298	0.0805	2.4550	1.8034	−0.1384	15.9894	0.0618
0.600	5.00	2.5470	2.1610	−0.1980	13.6900	0.1020	2.3580	1.7200	−0.1290	14.7300	0.0510
0.800	7.00	2.2410	1.5800	−0.1120	14.0300	0.0484	2.2020	1.5440	−0.1050	14.2000	0.0434
1.000	8.00	2.0980	1.4180	−0.0840	14.3500	0.0333	2.1040	1.4270	−0.0860	14.2000	0.0347
1.500	6.61	1.9841	1.2853	−0.0621	14.3908	0.0278	1.9347	1.2763	−0.0600	14.3463	0.0263
2.000	6.25	1.8876	1.1878	−0.0430	13.9796	0.0198	1.8409	1.1693	−0.0378	14.3787	0.0151
3.000	6.29	1.7416	1.0589	−0.0140	12.5838	0.0056	1.7129	1.0521	−0.0116	13.9009	0.0032
4.000	6.23	1.6476	0.9867	0.0040	23.1851	−0.0071	1.6267	0.9875	0.0040	13.1886	−0.0032
5.000	6.25	1.7045	0.9393	0.0173	14.2524	−0.0114	1.5648	0.9427	0.0159	14.6355	−0.0090
6.000	6.24	1.5229	0.9062	0.0270	13.8686	−0.0159	1.5128	0.9091	0.0269	13.5889	−0.0178
8.000	6.26	1.4354	0.8731	0.0366	15.6143	−0.0299	1.4300	0.8799	0.0349	12.0910	−0.0179
10.000	6.26	1.3697	0.8599	0.0406	12.8557	−0.0212	1.3742	0.8620	0.0395	14.3227	−0.0222
15.000	6.25	1.2747	0.8410	0.0436	15.2093	−0.0307	1.2801	0.8383	0.0475	15.6699	−0.0332

**Table 4:** (EBF and EABF) G–P fitting coefficients (b. c. a. X_k_ and d) of **CuZn05** sample.

E (MeV)	Z_eq_	EBF					EABF				
		b	c	a	X_k_	d	b	c	a	X_k_	d
0.015	10.49	1.0671	0.3869	0.2179	12.1098	−0.1119	1.0664	0.0985	0.1971	11.6964	−0.0931
0.020	10.80	1.1357	0.4043	0.2091	13.6052	−0.1121	1.1351	0.4154	0.1998	14.5453	−0.1093
0.030	11.20	1.3677	0.4737	0.1786	14.5553	−0.0941	1.3793	0.4665	0.1822	14.6027	−0.0957
0.040	11.46	1.7032	0.6129	0.1196	15.8297	−0.0609	1.7531	0.5953	0.1271	15.7675	−0.0666
0.050	11.64	2.2022	0.6704	0.1117	14.2060	−0.0620	2.3276	0.6629	0.1153	14.0830	−0.0669
0.060	11.78	2.5442	0.8183	0.0641	14.5671	−0.0516	2.8602	0.8097	0.0678	13.4014	−0.0476
0.080	11.99	2.9460	1.0399	0.0048	13.6992	−0.0200	3.7804	1.0590	−0.0012	14.3334	−0.0149
0.100	12.15	3.0617	1.2102	−0.0309	12.3990	−0.0061	4.2175	1.2634	−0.0444	12.7637	0.0055
0.150	12.39	2.9707	1.4156	−0.0683	17.7097	0.0112	4.1156	1.5317	−0.0923	13.6596	0.0293
0.200	12.37	2.8204	1.5059	−0.0827	16.3800	0.0167	3.6645	1.6465	−0.1099	13.8680	0.0375
0.300	13.20	2.5329	1.5015	−0.0835	16.3596	0.0165	3.1029	1.6248	−0.1071	14.2996	0.0337
0.400	12.71	2.4124	1.5099	−0.0872	16.1725	0.0202	2.7799	1.6085	−0.1058	14.6906	0.0326
0.500	12.76	2.2936	1.4813	−0.0844	16.3135	0.0204	2.5956	1.5442	−0.0963	15.2276	0.0276
0.600	13.10	2.1950	1.4456	−0.0805	17.1922	0.0226	2.4285	1.5186	−0.0955	14.8118	0.0318
0.800	12.73	2.0853	1.3885	−0.0731	16.0819	0.0210	2.2373	1.4416	−0.0845	14.7169	0.0288
1.000	12.79	1.9960	1.3388	−0.0666	15.8178	0.0219	2.1182	1.3700	−0.0734	15.0801	0.0259
1.500	8.91	1.9121	1.2545	−0.0543	14.7070	0.0212	1.9406	1.2583	−0.0553	14.2999	0.0217
2.000	7.29	1.8516	1.1723	−0.0388	14.3952	0.0163	1.8362	1.1732	−0.0391	14.1557	0.0163
3.000	7.24	1.7162	1.0587	−0.0132	13.6762	0.0040	1.7085	1.0557	−0.0125	12.9759	0.0036
4.000	7.24	1.6293	0.9892	0.0040	16.8470	−0.0050	1.6270	0.9815	0.0068	13.9861	−0.0070
5.000	7.19	1.5602	0.9414	0.0176	13.7491	−0.0125	1.5648	0.9332	0.0204	13.4637	−0.0144
6.000	7.20	1.5145	0.9043	0.0290	13.1225	−0.0195	1.4973	0.9290	0.0203	16.7108	−0.0177
8.000	7.17	1.4219	0.8877	0.0330	11.7862	−0.0165	1.4265	0.8740	0.0383	12.0935	−0.0224
10.000	7.17	1.3605	0.8728	0.0374	14.1703	−0.0227	1.3626	0.8695	0.0385	14.2524	−0.0232
15.000	7.15	1.2700	0.8412	0.0483	15.0384	−0.0347	1.2699	0.8404	0.0488	14.9603	−0.0357

**Table 5:** (EBF and EABF) G–P fitting coefficients (b. c. a. X_k_ and d) of **CuZn10** sample.

E (MeV)	Z_eq_	EBF					EABF				
		b	c	a	X_k_	d	b	c	a	X_k_	d
0.015	12.30	1.0345	0.3988	0.2095	14.0027	−0.1349	1.035	0.390	0.220	13.574	−0.145
0.020	12.69	1.0724	0.4005	0.2074	13.9690	−0.1116	1.076	0.372	0.224	14.220	−0.118
0.030	13.16	1.2155	0.4174	0.2011	14.9117	−0.1063	1.218	0.412	0.208	14.118	−0.114
0.040	13.48	1.4335	0.4820	0.1768	14.5599	−0.0977	1.454	0.474	0.180	14.722	−0.100
0.050	13.70	1.6763	0.6043	0.1245	15.4743	−0.0651	1.752	0.594	0.127	16.208	−0.069
0.060	13.86	1.9328	0.7013	0.0936	14.9754	−0.0524	2.212	0.614	0.135	39.767	−0.078
0.080	14.10	2.3799	0.8297	0.0607	14.4832	−0.0506	3.029	0.810	0.067	13.942	−0.051
0.100	14.26	2.5748	0.9969	0.0171	13.7637	−0.0326	3.642	0.999	0.016	13.748	0.008
0.150	14.54	2.6666	1.2322	−0.0338	11.0916	−0.0103	3.992	1.297	−0.050	17.236	0.005
0.200	14.66	2.6186	1.3376	−0.0513	8.5939	−0.0076	3.700	1.442	−0.075	15.567	0.016
0.300	14.71	2.4459	1.4433	−0.0737	18.5149	0.0128	3.126	1.544	−0.094	14.243	0.025
0.400	14.91	2.3276	1.4429	−0.0755	17.0509	0.0141	2.803	1.531	−0.093	15.172	0.026
0.500	15.06	2.2227	1.4320	−0.0758	16.3083	0.0156	2.591	1.502	−0.090	15.311	0.025
0.600	15.01	2.1469	1.4157	−0.0749	16.4573	0.0180	2.443	1.475	−0.087	14.947	0.026
0.800	15.18	2.0339	1.3709	−0.0701	15.8187	0.0198	2.246	1.407	−0.077	15.242	0.024
1.000	14.72	1.9648	1.3230	−0.0630	16.6856	0.0198	2.117	1.358	−0.071	15.002	0.024
1.500	9.77	1.8976	1.2455	−0.0520	14.9275	0.0193	1.939	1.256	−0.055	14.291	0.021
2.000	8.45	1.8298	1.1623	−0.0357	15.2335	0.0134	1.840	1.165	−0.036	14.642	0.014
3.000	8.17	1.7092	1.0523	−0.0110	12.7174	0.0019	1.710	1.052	−0.011	14.108	0.002
4.000	8.09	1.6234	0.9901	0.0041	20.2274	−0.0071	1.621	0.986	0.006	12.977	−0.007
5.000	8.08	1.5525	0.9473	0.0160	14.4433	−0.0114	1.556	0.942	0.018	13.262	−0.013
6.000	8.07	1.5047	0.9169	0.0252	15.5315	−0.0229	1.505	0.907	0.029	15.103	−0.026
8.000	8.02	1.4169	0.8911	0.0330	12.3083	−0.0194	1.411	0.896	0.031	12.330	−0.017
10.000	8.03	1.3579	0.8721	0.0391	13.9794	−0.0253	1.356	0.867	0.041	13.900	−0.027
15.000	7.99	1.2651	0.8420	0.0500	15.0301	−0.0386	1.259	0.848	0.048	14.753	−0.036

**Table 6:** (EBF and EABF) G–P fitting coefficients (b. c. a. X_k_ and d) of **CuZn15** sample.

E (MeV)	Z_eq_	EBF					EABF				
		b	c	a	X_k_	d	b	c	a	X_k_	d
0.015	13.59	1.0254	0.3672	0.2394	13.4062	−0.1628	1.0248	0.3944	0.2102	12.3559	−0.1150
0.020	14.01	1.0509	0.4261	0.1789	17.5870	−0.1074	1.0509	0.4258	0.1792	17.5836	−0.1076
0.030	14.54	1.1583	0.3945	0.2144	14.2188	−0.1156	1.1588	0.3923	0.2172	14.1113	−0.1202
0.040	14.87	1.3232	0.8769	0.1941	14.4170	−0.1082	1.3314	0.4411	0.1934	14.6535	−0.1067
0.050	15.10	1.5236	0.5167	0.1634	14.6819	−0.0910	1.5632	0.5205	0.1582	15.1865	−0.0851
0.060	15.28	1.7180	0.6168	0.1235	14.7374	−0.0676	1.9127	0.5472	0.1589	14.1375	−0.0836
0.080	15.53	2.0764	0.7602	0.0787	13.9917	−0.0478	2.6399	0.6844	0.1120	13.3225	−0.0743
0.100	15.68	2.3247	0.8902	0.0448	13.3232	−0.0429	3.2618	0.8666	0.0526	13.6278	−0.0497
0.150	15.94	2.4993	1.1370	−0.0148	12.5436	−0.0192	3.8420	1.1705	−0.0242	13.3164	−0.0100
0.200	16.11	2.4908	1.2633	−0.0379	11.0356	−0.0127	3.6751	1.3306	−0.0543	19.2665	0.0042
0.300	16.41	2.3837	1.3528	−0.0543	8.0921	−0.0100	3.1532	1.4525	−0.0773	17.2775	0.0177
0.400	16.43	2.2735	1.4000	−0.0672	17.6973	0.0099	2.8375	1.4639	−0.0802	16.8703	0.0182
0.500	16.39	2.1896	1.4075	−0.0716	17.4940	0.0154	2.6023	1.4662	−0.0830	15.8316	0.0213
0.600	16.67	2.1187	1.3840	−0.0686	18.3554	0.0156	2.4482	1.4394	−0.0799	16.1414	0.0216
0.800	16.66	2.0170	1.3489	−0.0653	16.5729	0.0166	2.2506	1.3881	−0.0736	15.4794	0.0217
1.000	16.62	1.9427	1.3098	−0.0604	15.9077	0.0171	2.1262	1.3313	−0.0660	15.3990	0.0209
1.500	10.92	1.8801	1.2347	−0.0492	15.1934	0.0169	1.9362	1.2532	−0.0540	14.2807	0.0205
2.000	9.41	1.8156	1.1589	−0.0350	14.9053	0.0129	1.8421	1.1604	−0.0355	14.6863	0.0134
3.000	8.99	1.7011	1.0535	−0.0110	12.0003	0.0016	1.7085	1.0513	−0.0103	12.9985	0.0007
4.000	8.91	1.6179	0.9907	0.0047	17.9186	−0.0076	1.6186	0.9850	0.0070	13.0340	−0.0087
5.000	8.88	1.5474	0.9499	0.0160	14.6750	−0.0142	1.5514	0.9433	0.0183	13.0806	−0.0135
6.000	8.84	1.5015	0.9161	0.0269	14.3029	−0.0235	1.4975	0.9132	0.0274	15.3593	−0.0263
8.000	8.80	1.4137	0.8943	0.0330	12.6694	−0.0214	1.4041	0.8981	0.0316	12.3150	−0.0186
10.000	8.80	1.3547	0.8732	0.0402	13.7516	−0.0277	1.3465	0.8750	0.0395	13.9089	−0.0277
15.000	8.79	1.2644	0.8364	0.0541	14.8293	−0.0439	1.2490	0.8610	0.0450	14.7411	−0.0161

**Table 7:** (EBF and EABF) G–P fitting coefficients (b. c. a. X_k_ and d) of **CuZn20** sample.

E (MeV)	Z_eq_	EBF					EABF				
		b	c	a	X_k_	d	b	c	a	X_k_	d
0.015	14.59	1.0200	0.3783	0.2321	11.9979	−0.1485	1.0196	0.4048	0.2032	11.6685	−0.1077
0.020	15.04	1.0409	0.4279	0.1774	14.4564	−0.0908	1.0410	0.4062	0.1971	14.2506	−0.1145
0.030	15.59	1.1254	0.3898	0.2142	14.0982	−0.1153	1.1254	0.3882	0.2166	13.8820	−0.1184
0.040	15.93	1.2608	0.4528	0.2034	14.5887	−0.1123	1.2706	0.4073	0.2117	14.7440	−0.1228
0.050	16.17	1.4283	0.4820	0.1784	14.5750	−0.1003	1.4632	0.4745	0.1809	14.6741	−0.1010
0.060	16.36	1.6014	0.5632	0.1453	14.5774	−0.0811	1.6933	0.5650	0.1409	15.2738	−0.0771
0.080	16.62	1.8920	0.7284	0.0859	14.7435	−0.0503	2.4025	0.6134	0.1401	13.2621	−0.0879
0.100	16.79	2.1427	0.8459	0.0543	13.6113	−0.0428	2.9886	0.7830	0.0792	13.4774	−0.0628
0.150	17.08	2.3611	1.0817	−0.0041	13.0417	−0.0223	3.6821	1.0778	−0.0026	13.2058	−0.0218
0.200	17.21	2.4051	1.2107	−0.0278	11.6463	−0.0166	3.6297	1.2521	−0.0380	15.0416	−0.0071
0.300	17.23	2.3392	1.3314	−0.0510	8.5743	−0.0103	3.1573	1.4134	−0.0703	19.3959	0.0150
0.400	17.54	2.2453	1.3586	−0.0578	10.9611	−0.0037	2.8314	1.4368	−0.0758	16.7486	0.0161
0.500	17.52	2.1579	1.3865	−0.0676	20.8720	0.0172	2.6113	1.4362	−0.0773	16.7822	0.0185
0.600	17.56	2.0979	1.3738	−0.0668	18.7309	0.0153	2.4535	1.4195	−0.0759	16.8040	0.0194
0.800	17.70	1.9989	1.3427	−0.0643	16.5470	0.0166	2.2486	1.3778	−0.0716	15.6293	0.0205
1.000	17.55	1.9305	1.3051	−0.0594	15.9916	0.0167	2.1206	1.3331	−0.0660	15.0770	0.0208
1.500	12.25	1.8639	1.2322	−0.0487	14.9052	0.0169	1.9400	1.2784	−0.0527	14.5643	0.0200
2.000	10.24	1.8044	1.1562	−0.0344	14.6466	0.0125	1.8437	1.1569	−0.0347	14.7210	0.0129
3.000	9.86	1.6933	1.0546	−0.0110	11.2965	0.0013	1.7074	1.0507	−0.0097	11.9092	−0.0002
4.000	9.67	1.6133	0.9912	0.0052	15.9460	−0.0081	1.6168	0.9842	0.0078	13.0832	−0.0100
5.000	9.63	1.5431	0.9522	0.0160	14.8733	−0.0166	1.5479	0.9443	0.0186	12.9255	−0.0141
6.000	9.58	1.4988	0.9153	0.0284	13.2343	−0.0239	1.4908	0.9190	0.0262	15.5826	−0.0266
8.000	9.56	1.4108	0.8972	0.0330	12.9880	−0.0231	1.3981	0.8999	0.0321	12.3020	−0.0203
10.000	9.54	1.3519	0.8742	0.0412	13.5487	−0.0298	1.3383	0.8819	0.0382	13.9165	−0.0280
15.000	9.51	1.2639	0.8317	0.0576	14.6614	−0.0482	1.2406	0.8719	0.0426	14.7337	0.0010

## Data Availability

The data presented in this study are available on request from the corresponding author.

## References

[1] McCaffrey J.P., Shen H., Downton B., Mainegra-Hing E. (2007). Radiation attenuation by lead and nonlead materials used in radiation shielding garments. Med. Phys..

[2] Singh J., Kumar V., Vermani Y.K., Al-Buriahi M., Alzahrani J.S., Singh T. (2021). Fabrication and characterization of barium based bioactive glasses in terms of physical, structural, mechanical and radiation shielding properties. Ceram. Int..

[3] More C.V., Alsayed Z., Badawi M.S., Thabet A.A., Pawar P.P. (2021). Polymeric composite materials for radiation shielding: A review. Environ. Chem. Lett..

[4] Tekin H.O. (2016). MCNP-X Monte Carlo Code Application for Mass Attenuation Coefficients of Concrete at Different Energies by Modeling 3 × 3 Inch NaI(Tl) Detector and Comparison with XCOM and Monte Carlo Data. Sci. Technol. Nucl. Install..

[5] Tekin H., Singh V., Manici T. (2017). Effects of micro-sized and nano-sized WO_3_ on mass attenauation coefficients of concrete by using MCNPX code. Appl. Radiat. Isot..

[6] Dilsiz K., Ogul H., Akman F., Agar O., Kacal M.R., Polat H., Dursun İ. (2021). Evaluation of CdS doped polyester composites regarding gamma and neutron shielding properties. Prog. Nucl. Energy.

[7] Al-Buriahi M., El-Agawany F., Sriwunkum C., Akyildirim H., Arslan H., Tonguc B., El-Mallawany R., Rammah Y. (2020). Influence of Bi_2_O_3_/PbO on nuclear shielding characteristics of lead-zinc-tellurite glasses. Phys. B Condens. Matter.

[8] Al-Buriahi M.S., Sriwunkum C., Arslan H., Tonguc B.T., Bourham M.A. (2020). Investigation of barium borate glasses for radiation shielding applications. Appl. Phys. A.

[9] Abouhaswa A.S., Al-Buriahi M.S., Chalermpon M., Rammah Y.S. (2020). Influence of ZrO_2_ on gamma shielding properties of lead borate glasses. Appl. Phys. A.

[10] Akman F., Sayyed M., Kaçal M., Tekin H. (2019). Investigation of photon shielding performances of some selected alloys by experimental data, theoretical and MCNPX code in the energy range of 81 keV–1333 keV. J. Alloys Compd..

[11] Agar O., Sayyed M., Akman F., Tekin H.O., Kaçal M. (2019). An extensive investigation on gamma ray shielding features of Pd/Ag-based alloys. Nucl. Eng. Technol..

[12] Tekin H., Kilicoglu O. (2020). The influence of gallium (Ga) additive on nuclear radiation shielding effectiveness of Pd/Mn binary alloys. J. Alloys Compd..

[13] Mesbahi A., Ghiasi H. (2018). Shielding properties of the ordinary concrete loaded with micro- and nano-particles against neutron and gamma radiations. Appl. Radiat. Isot..

[14] Mesbahi A., Azarpeyvand A.-A., Shirazi A. (2011). Photoneutron production and backscattering in high density concretes used for radiation therapy shielding. Ann. Nucl. Energy.

[15] Akman F., Kaçal M., Almousa N., Sayyed M., Polat H. (2020). Gamma-ray attenuation parameters for polymer composites reinforced with BaTiO_3_ and CaWO_4_ compounds. Prog. Nucl. Energy.

[16] AL-Dhuhaibat M.J.R. (2015). Study of the shielding properties for some composite materials manufactured from polymer epoxy supported by cement, aluminum, iron and lead against gamma rays of the cobalt radioactive source (Co-60). Int. J. Appl. Innov. Eng. Manag..

[17] (2002). RSICC Computer Code Collection. MCNPX User’s Manual Version 2.4.0. Monte Carlo N-Particle Transport Code System for Multiple and High Energy Applications. http://www.nea.fr/abs/html/ccc-0715.html.

[18] National Nuclear Data Center (NNDC) in Brookhaven National Laboratory, National Nuclear Data Center (NNDC) in Brookhaven National Laboratory (n.d.). https://www.nndc.bnl.gov/nudat2/.

[19] (2018). Maestro, No Title. http://www.Ortec-Online.Com/Download/Maest.

[20] Agar O., Boztosun I., Segebade C. (2017). Multielemental analysis of some soils in Karaman by PAA using a cLINAC. Appl. Radiat. Isot..

[21] Al-Buriahi M.S., Tonguc B.T. (2020). Mass attenuation coefficients, effective atomic numbers and electron densities of some contrast agents for computed tomography. Radiat. Phys. Chem..

[22] Issa S.A., Zakaly H.M., Pyshkina M., Mostafa M.Y., Rashad M., Soliman T. (2021). Structure, optical, and radiation shielding properties of PVA–BaTiO_3_ nanocomposite films: An experimental investigation. Radiat. Phys. Chem..

[23] Harima Y., Sakamoto Y., Tanaka S., Kawai M. (1986). Validity of the Geometric-Progression Formula in Approximating Gamma-Ray Buildup Factors. Nucl. Sci. Eng..

[24] Kurtuluş R., Kavas T., Agar O., Turhan M.F., Kaçal M.R., Dursun I., Akman F. (2021). Study on recycled Er-incorporated waste CRT glasses for photon and neutron shielding. Ceram. Int..

[25] Kumar A. (2017). Gamma ray shielding properties of PbO-Li_2_O-B_2_O_3_ glasses. Radiat. Phys. Chem..

[26] Ziegler J.F., Ziegler M., Biersack J. (2010). SRIM—The stopping and range of ions in matter. Nucl. Instrum. Methods Phys. Res. B.

[27] Belgin E.E., Aycik G., Kalemtas A., Pelit A., Dilek D., Kavak M. (2015). Preparation and characterization of a novel ionizing electromagnetic radiation shielding material: Hematite filled polyester based composites. Radiat. Phys. Chem..

[28] Lamarash J.R., Baratta A.J. (2001). Introduction to Nuclear Engineering.

[29] Ahmed B., Shah G., Malik A.H., Rizwan M. (2020). Gamma-ray shielding characteristics of flexible silicone tungsten composites. Appl. Radiat. Isot..

[30] Alsayed Z., Badawi M.S., Awad R., El-Khatib A.M., Thabet A.A. (2020). Investigation of γ-ray attenuation coefficients, effective atomic number and electron density for ZnO/HDPE composite. Phys. Scr..

